# Supplementary material for: BONCAT-Live for isolation and cultivation of active environmental microbes
Source: bioRxiv. 2025 May 14:2025.05.14.654084. Preprint. [Version 1] doi: 10.1101/2025.05.14.654084 (PMC12132240; doi:10.1101/2025.05.14.654084)
Supplement: Supplement 1 [file media-1.pdf]

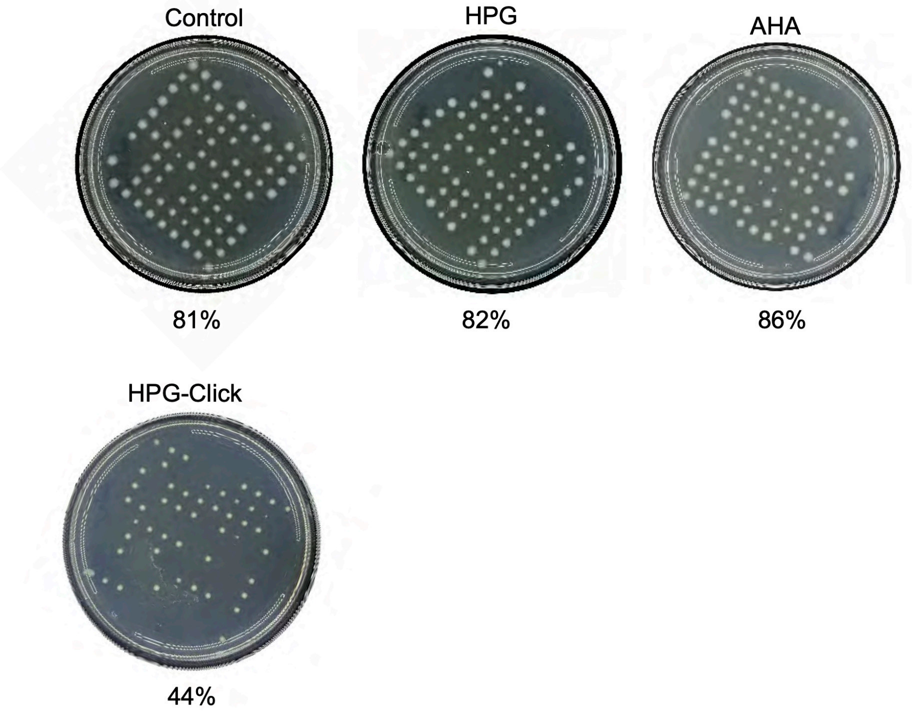

Figure S1. Viability tests for flow sorted *Pseudomonas* sp GH41. On each test, 100 particles were individually deposited on R2A agar plate, followed by incubation (2 days at 25C). Control, untreated cells grown in R2A*; HPG and AHA, cells grown in R2A* supplemented with HPG or AHA, respectively; HPG-Click, cells grown in R2A* supplemented with HPG that were click-labeled using copper chemistry protocol. The percentages indicate number of colonies.
