# Supplementary material for: BONCAT-Live for isolation and cultivation of active environmental microbes
Source: bioRxiv. 2025 May 14:2025.05.14.654084. Preprint. [Version 1] doi: 10.1101/2025.05.14.654084 (PMC12132240; doi:10.1101/2025.05.14.654084)
Supplement: Supplement 2 [file media-2.pdf]

Relative taxon abundance in rhizosphere soil and in populations sorted follwing BONCAT incubations.

R, sorted based on red fluoresence (DNA stain). G, sorted based on A488 click label

Taxon	Rhizosphere	NoHPG R	HPG R	HPG Low G	HPG High G	HPG Salicin R	HPG Salicin G	HPG Malate R	HPG Malate G
Acidobacteriota;c__Acidobacteriae;o__Acidobacteriales	0.18	0.00	0.00	0.00	0.00	0.00	0.00	0.00	0.00
Acidobacteriota;c__Acidobacteriae;o__Acidobacteriales;f__Acidobacteriaceae_(Subgroup_1)	0.13	0.18	0.10	0.01	0.00	0.00	0.01	0.00	0.00
Acidobacteriota;c__Acidobacteriae;o__Bryobacterales;f__Bryobacteraceae;g__Bryobacter	0.32	0.00	0.00	0.00	0.00	0.00	0.00	0.00	0.00
Acidobacteriota;c__Acidobacteriae;o__Solibacterales;f__Solibacteraceae;g__Candidatus_Solibacter	0.84	0.00	0.00	0.02	0.00	0.00	0.00	0.00	0.00
Acidobacteriota;c__Acidobacteriae;o__Subgroup_2;f__Subgroup_2;g__Subgroup_2	0.37	0.00	0.00	0.00	0.00	0.00	0.00	0.00	0.00
Acidobacteriota;c__Blastocatellia;o__11-24;f__11-24;g__11-24	0.69	0.00	0.00	0.00	0.00	0.00	0.00	0.00	0.00
Acidobacteriota;c__Blastocatellia;o__Blastocatellales;f__Blastocatellaceae	0.17	0.00	0.00	0.00	0.00	0.00	0.00	0.00	0.00
Acidobacteriota;c__Blastocatellia;o__Elev-16S-573;f__Elev-16S-573;g__Elev-16S-573	0.10	0.00	0.00	0.00	0.00	0.00	0.00	0.00	0.00
Acidobacteriota;c__Blastocatellia;o__Pyrinomonadales;f__Pyrinomonadaceae;g__RB41	1.19	0.00	0.00	0.00	0.00	0.00	0.00	0.00	0.00
Acidobacteriota;c__Holophagae;o__Subgroup_7;f__Subgroup_7;g__Subgroup_7	0.54	0.00	0.00	0.00	0.00	0.00	0.00	0.00	0.00
Acidobacteriota;c__Subgroup_22;o__Subgroup_22;f__Subgroup_22;g__Subgroup_22	0.19	0.00	0.00	0.00	0.00	0.00	0.00	0.00	0.00
Acidobacteriota;c__Subgroup_25;o__Subgroup_25;f__Subgroup_25;g__Subgroup_25	0.13	0.00	0.00	0.00	0.00	0.00	0.00	0.00	0.00
Acidobacteriota;c__Subgroup_5;o__Subgroup_5;f__Subgroup_5;g__Subgroup_5	0.28	0.00	0.00	0.00	0.00	0.00	0.00	0.00	0.00
Acidobacteriota;c__Vicinamibacteria;o__Subgroup_17;f__Subgroup_17;g__Subgroup_17	0.79	0.00	0.05	0.00	0.00	0.04	0.00	0.03	0.00
Acidobacteriota;c__Vicinamibacteria;o__Vicinamibacterales;f__uncultured;g__uncultured	7.52	0.31	0.04	0.03	0.07	0.20	0.00	0.01	0.02
Acidobacteriota;c__Vicinamibacteria;o__Vicinamibacterales;f__Vicinamibacteraceae;g__uncultured	0.21	0.00	0.00	0.00	0.00	0.00	0.00	0.00	0.00
Acidobacteriota;c__Vicinamibacteria;o__Vicinamibacterales;f__Vicinamibacteraceae;g__Vicinamibacteraceae	1.13	0.13	0.06	0.00	0.00	0.05	0.00	0.00	0.01
Actinobacteriota;c__Acidimicrobiia;o__IMCC26256;f__IMCC26256;g__IMCC26256	1.60	0.00	0.00	0.00	0.00	0.00	0.00	0.00	0.00
Actinobacteriota;c__Acidimicrobiia;o__Microtrichales	0.19	0.00	0.00	0.00	0.00	0.00	0.00	0.00	0.02
Actinobacteriota;c__Acidimicrobiia;o__Microtrichales;f__Ilumatobacteraceae	0.12	0.05	0.00	0.00	0.00	0.00	0.00	0.00	0.00
Actinobacteriota;c__Acidimicrobiia;o__Microtrichales;f__Ilumatobacteraceae;g__CL500-29_marine_group	0.23	0.00	0.02	0.00	0.00	0.04	0.00	0.01	0.00
Actinobacteriota;c__Acidimicrobiia;o__Microtrichales;f__Ilumatobacteraceae;g__uncultured	0.58	0.00	0.00	0.00	0.00	0.00	0.00	0.00	0.00
Actinobacteriota;c__Acidimicrobiia;o__Microtrichales;f__uncultured;g__uncultured	0.32	0.00	0.00	0.00	0.00	0.00	0.00	0.00	0.00
Actinobacteriota;c__Actinobacteria	1.41	0.12	0.79	1.01	0.00	2.82	4.78	0.10	0.00
Actinobacteriota;c__Actinobacteria;o__Corynebacteriales;f__Mycobacteriaceae;g__Mycobacterium	0.65	0.27	0.38	2.12	0.00	0.09	0.32	0.06	0.04
Actinobacteriota;c__Actinobacteria;o__Frankiales;f__Acidothermaceae;g__Acidothermus	0.65	0.00	0.00	0.00	0.00	0.00	0.00	0.00	0.00
Actinobacteriota;c__Actinobacteria;o__Frankiales;f__Frankiaceae;g__Jatrophihabitans	0.22	0.00	0.00	0.00	0.00	0.00	0.00	0.00	0.00
Actinobacteriota;c__Actinobacteria;o__Frankiales;f__Nakamurellaceae;g__Nakamurella	0.04	0.00	0.00	0.00	0.00	0.09	0.13	0.00	0.00
Actinobacteriota;c__Actinobacteria;o__Frankiales;f__Sporichthyaceae	0.13	0.00	0.00	0.00	0.00	0.00	0.00	0.00	0.00
Actinobacteriota;c__Actinobacteria;o__Micrococcales;f__Microbacteriaceae	0.05	0.36	1.87	0.79	0.00	1.54	0.07	0.13	0.37
Actinobacteriota;c__Actinobacteria;o__Micrococcales;f__Microbacteriaceae	0.11	1.43	4.39	0.94	0.05	2.57	0.15	0.42	0.91
Actinobacteriota;c__Actinobacteria;o__Micrococcales;f__Microbacteriaceae;g__Agromyces	0.04	0.10	0.00	0.00	0.00	0.00	0.00	0.00	0.00
Actinobacteriota;c__Actinobacteria;o__Micrococcales;f__Microbacteriaceae;g__Microbacterium	0.02	0.97	2.52	0.15	0.05	1.03	0.07	0.29	0.54
Actinobacteriota;c__Actinobacteria;o__Micrococcales;f__Micrococcaceae	0.06	1.87	2.11	2.12	0.07	20.02	25.22	0.89	0.21
Actinobacteriota;c__Actinobacteria;o__Micromonosporales;f__Micromonosporaceae	0.65	0.02	0.03	0.00	0.03	0.00	0.00	0.00	0.00
Actinobacteriota;c__Actinobacteria;o__Micromonosporales;f__Micromonosporaceae;g__Dactylosporangium	0.11	0.00	0.00	0.00	0.00	0.00	0.00	0.00	0.00
Actinobacteriota;c__Actinobacteria;o__Propionibacteriales;f__Nocardioidaceae	0.29	0.20	0.00	0.00	0.00	0.39	0.05	0.00	0.00
Actinobacteriota;c__Actinobacteria;o__Propionibacteriales;f__Nocardioidaceae	1.42	0.75	2.93	0.16	0.08	1.31	0.31	0.34	0.08
Actinobacteriota;c__Actinobacteria;o__Propionibacteriales;f__Nocardioidaceae;g__Aeromicrobium	0.00	0.07	1.17	0.04	0.00	0.00	0.00	0.16	0.03
Actinobacteriota;c__Actinobacteria;o__Propionibacteriales;f__Nocardioidaceae;g__Kribbella	0.12	0.00	0.00	0.00	0.00	0.00	0.00	0.00	0.00
Actinobacteriota;c__Actinobacteria;o__Propionibacteriales;f__Nocardioidaceae;g__Marmoricola	0.04	0.05	0.04	0.00	0.00	0.00	0.00	0.00	0.00
Actinobacteriota;c__Actinobacteria;o__Propionibacteriales;f__Nocardioidaceae;g__Nocardioides	0.97	0.43	1.71	0.12	0.08	0.92	0.26	0.18	0.05
Actinobacteriota;c__Actinobacteria;o__Propionibacteriales;f__Propionibacteriaceae;g__Microlunatus	0.47	0.00	0.00	0.00	0.00	0.00	0.04	0.00	0.00
Actinobacteriota;c__Actinobacteria;o__Pseudonocardiales;f__Pseudonocardiaceae;g__Pseudonocardia	0.41	0.00	0.00	0.00	0.00	0.00	0.00	0.00	0.00
Actinobacteriota;c__Actinobacteria;o__Streptomycetales;f__Streptomycetaceae	0.24	0.00	0.00	0.00	0.07	0.35	0.65	0.00	0.00
Actinobacteriota;c__Actinobacteria;o__Streptomycetales;f__Streptomycetaceae;g__Streptomyces	0.27	0.00	0.00	0.00	0.00	0.00	0.00	0.00	0.00
Actinobacteriota;c__Actinobacteria;o__Streptosporangiales;f__Thermomonosporaceae;g__Actinoallomurus	0.13	0.00	0.00	0.00	0.00	0.00	0.00	0.00	0.00
Actinobacteriota;c__MB-A2-108;o__MB-A2-108;f__MB-A2-108;g__MB-A2-108	1.97	0.00	0.00	0.00	0.00	0.00	0.00	0.00	0.00
Actinobacteriota;c__Rubrobacteria;o__Rubrobacterales;f__Rubrobacteriaceae;g__Rubrobacter	0.23	0.00	0.00	0.00	0.00	0.00	0.00	0.00	0.01
Actinobacteriota;c__Thermoleophilia;o__Gaiellales	4.51	0.02	0.01	0.00	0.00	0.00	0.00	0.00	0.00
Actinobacteriota;c__Thermoleophilia;o__Gaiellales;f__Gaiellaceae;g__Gaiella	1.31	0.00	0.00	0.00	0.00	0.11	0.00	0.00	0.00
Actinobacteriota;c__Thermoleophilia;o__Gaiellales;f__uncultured;g__uncultured	2.08	0.00	0.00	0.00	0.00	0.00	0.00	0.01	0.00
Actinobacteriota;c__Thermoleophilia;o__Solirubrobacterales;f__67-14;g__67-14	2.48	0.03	0.00	0.00	0.00	0.00	0.00	0.00	0.02
Actinobacteriota;c__Thermoleophilia;o__Solirubrobacterales;f__Solirubrobacteraceae	0.63	0.00	0.00	0.00	0.00	0.00	0.00	0.00	0.00
Actinobacteriota;c__Thermoleophilia;o__Solirubrobacterales;f__Solirubrobacteraceae;g__Conexibacter	0.83	0.00	0.00	0.00	0.00	0.00	0.01	0.00	0.00
Actinobacteriota;c__Thermoleophilia;o__Solirubrobacterales;f__Solirubrobacteraceae;g__Solirubrobacter	0.29	0.00	0.00	0.00	0.00	0.13	0.00	0.00	0.00
Actinobacteriota;c__Thermoleophilia;o__uncultured;f__uncultured;g__uncultured	0.28	0.00	0.00	0.00	0.00	0.00	0.00	0.00	0.00
Armatimonadota;c__Armatimonadia;o__Armatimonadales;f__Armatimonadales;g__Armatimonadales	0.03	0.00	0.02	0.30	0.00	0.03	0.01	0.00	0.07
Armatimonadota;c__Chthonomonadetes;o__Chthonomonadales;f__Chthonomonadaceae;g__Chthonomonas	0.04	0.00	0.02	0.04	0.00	0.00	0.00	0.00	0.00
Armatimonadota;c__Fimbriimonadia;o__Fimbriimonadales;f__Fimbriimonadaceae;g__Fimbriimonadaceae	0.02	0.00	0.08	0.01	0.00	0.00	0.00	0.00	0.00
Bacteroidota;c__Bacteroidia;o__Bacteroidales;f__Marinilabiliaceae;g__[Cytophaga]_xylanolytica_group	0.00	0.00	0.00	0.00	0.00	0.00	0.00	0.00	0.00
Bacteroidota;c__Bacteroidia;o__Chitinophagales;f__Chitinophagaceae	0.35	0.66	0.17	0.01	0.04	0.09	0.72	0.20	0.02
Bacteroidota;c__Bacteroidia;o__Chitinophagales;f__Chitinophagaceae	0.63	0.72	0.72	0.48	0.06	0.40	1.30	0.64	0.97
Bacteroidota;c__Bacteroidia;o__Chitinophagales;f__Chitinophagaceae;g__Aurantisolimonas	0.02	0.00	0.00	0.00	0.00	0.00	0.03	0.00	0.21
Bacteroidota;c__Bacteroidia;o__Chitinophagales;f__Chitinophagaceae;g__Chitinophaga	0.01	0.01	0.00	0.22	0.00	0.21	0.08	0.00	0.46
Bacteroidota;c__Bacteroidia;o__Chitinophagales;f__Chitinophagaceae;g__Edaphobaculum	0.06	0.00	0.05	0.04	0.03	0.00	0.00	0.00	0.00
Bacteroidota;c__Bacteroidia;o__Chitinophagales;f__Chitinophagaceae;g__Ferruginibacter	0.07	0.00	0.00	0.01	0.00	0.00	0.07	0.00	0.02
Bacteroidota;c__Bacteroidia;o__Chitinophagales;f__Chitinophagaceae;g__Heliimonas	0.00	0.05	0.16	0.20	0.00	0.05	0.41	0.00	0.02
Bacteroidota;c__Bacteroidia;o__Chitinophagales;f__Chitinophagaceae;g__Taibaiella	0.00	0.00	0.34	0.00	0.00	0.04	0.00	0.44	0.24
Bacteroidota;c__Bacteroidia;o__Chitinophagales;f__Chitinophagaceae;g__uncultured	0.12	0.00	0.00	0.00	0.00	0.00	0.00	0.00	0.00
Bacteroidota;c__Bacteroidia;o__Cytophagales;f__Spirosomaceae;g__Dyadobacter	0.00	0.03	0.08	0.00	0.00	0.19	0.38	0.15	2.15
Bacteroidota;c__Bacteroidia;o__Flavobacteriales;f__Flavobacteriaceae;g__Flavobacterium	0.27	1.68	6.38	0.71	0.72	0.07	0.00	4.13	2.52
Bacteroidota;c__Bacteroidia;o__Flavobacteriales;f__Weeksellaceae	0.00	0.00	0.00	0.00	0.00	0.00	0.00	0.05	0.53
Bacteroidota;c__Bacteroidia;o__Flavobacteriales;f__Weeksellaceae;g__Chryseobacterium	0.00	0.49	0.64	0.23	0.09	0.35	0.13	1.38	0.76
Bacteroidota;c__Bacteroidia;o__Sphingobacteriales;f__AKYH767;g__AKYH767	0.13	0.00	0.00	0.00	0.00	0.00	0.00	0.00	0.00
Bacteroidota;c__Bacteroidia;o__Sphingobacteriales;f__Sphingobacteriaceae	0.00	0.00	0.02	0.02	0.00	0.00	0.11	0.11	0.27
Bacteroidota;c__Bacteroidia;o__Sphingobacteriales;f__Sphingobacteriaceae;g__Mucilaginibacter	0.00	2.99	6.51	4.06	0.38	1.23	1.16	0.40	1.28
Bacteroidota;c__Bacteroidia;o__Sphingobacteriales;f__Sphingobacteriaceae;g__Pedobacter	0.00	0.80	3.93	0.67	0.12	0.28	0.15	4.10	3.27
Bdellovibrionota;c__Bdellovibrionia;o__Bacteriovoracales;f__Bacteriovoracaceae;g__Bacteriovorax	0.00	0.11	0.03	0.11	0.00	0.00	0.00	0.06	0.01
Bdellovibrionota;c__Bdellovibrionia;o__Bdellovibrionales;f__Bdellovibrionaceae;g__Bdellovibrio	0.10	0.00	0.00	0.00	0.00	0.00	0.00	0.00	0.00
Bdellovibrionota;c__Oligoflexia;o__0319-6G20;f__0319-6G20;g__0319-6G20	0.18	0.00	0.00	0.00	0.00	0.00	0.00	0.00	0.00
Chloroflexi;c__Anaerolineae;o__Anaerolineales;f__Anaerolineaceae;g__Anaerolinea	0.26	0.00	0.00	0.00	0.00	0.00	0.00	0.00	0.00
Chloroflexi;c__Anaerolineae;o__Anaerolineales;f__Anaerolineaceae;g__uncultured	0.13	0.00	0.00	0.00	0.00	0.00	0.00	0.00	0.00
Chloroflexi;c__Anaerolineae;o__Anaerolineales;f__Anaerolineaceae;g__UTCFX1	0.12	0.00	0.00	0.00	0.00	0.00	0.00	0.00	0.00
Chloroflexi;c__Anaerolineae;o__Caldilineales;f__Caldilineaceae;g__uncultured	0.09	0.12	0.00	0.00	0.00	0.00	0.00	0.00	0.00
Chloroflexi;c__Anaerolineae;o__RBG-13-54-9;f__RBG-13-54-9;g__RBG-13-54-9	0.36	0.00	0.00	0.00	0.00	0.00	0.00	0.00	0.00
Chloroflexi;c__Anaerolineae;o__SBR1031;f__A4b;g__A4b	0.11	0.01	0.00	0.05	0.00	0.00	0.14	0.00	0.01
Chloroflexi;c__Anaerolineae;o__SBR1031;f__SBR1031;g__SBR1031	0.15	0.00	0.00	0.00	0.00	0.00	0.00	0.00	0.00
Chloroflexi;c__Chloroflexia;o__Chloroflexales;f__Roseiflexaceae;g__uncultured	0.12	0.00	0.00	0.00	0.00	0.00	0.00	0.00	0.00
Chloroflexi;c__Chloroflexia;o__Thermomicrobiales;f__JG30-KF-CM45;g__JG30-KF-CM45	0.63	0.00	0.00	0.00	0.00	0.08	0.00	0.00	0.00
Chloroflexi;c__Dehalococcoidia;o__S085;f__S085;g__S085	0.15	0.00	0.00	0.00	0.00	0.00	0.00	0.00	0.00
Chloroflexi;c__Gitt-GS-136;o__Gitt-GS-136;f__Gitt-GS-136;g__Gitt-GS-136	0.12	0.00	0.00	0.00	0.00	0.00	0.00	0.00	0.00
Chloroflexi;c__JG30-KF-CM66;o__JG30-KF-CM66;f__JG30-KF-CM66;g__JG30-KF-CM66	0.07	0.02	0.00	0.00	0.00	0.03	0.49	0.00	0.00
Chloroflexi;c__KD4-96;o__KD4-96;f__KD4-96;g__KD4-96	3.04	0.01	0.04	0.00	0.00	0.00	0.00	0.03	0.00
Chloroflexi;c__Ktedonobacteria;o__C0119;f__C0119;g__C0119	0.25	0.00	0.00	0.00	0.00	0.00	0.00	0.00	0.00
Chloroflexi;c__Ktedonobacteria;o__Ktedonobacterales;f__JG30-KF-AS9;g__JG30-KF-AS9	0.28	0.00	0.00	0.00	0.00	0.00	0.00	0.00	0.00
Chloroflexi;c__Ktedonobacteria;o__Ktedonobacterales;f__Ktedonobacteraceae	0.10	0.00	0.00	0.00	0.00	0.00	0.00	0.00	0.00
Chloroflexi;c__Ktedonobacteria;o__Ktedonobacterales;f__Ktedonobacteraceae;g__uncultured	0.10	0.00	0.00	0.00	0.00	0.00	0.00	0.00	0.00
Chloroflexi;c__TK10;o__TK10;f__TK10;g__TK10	0.85	0.00	0.00	0.00	0.00	0.09	0.00	0.00	0.00
Desulfobacterota;c__Desulfuromonadia;o__Geobacterales;f__Geobacteraceae	0.14	0.00	0.00	0.00	0.00	0.00	0.00	0.00	0.00
Desulfobacterota;c__uncultured;o__uncultured;f__uncultured;g__uncultured	0.21	0.00	0.00	0.00	0.00	0.00	0.00	0.00	0.00
Firmicutes;c__Bacilli;o__Bacillales;f__Bacillaceae;g__Bacillus	0.74	0.00	0.00	0.00	0.00	0.00	0.10	0.00	0.00
Firmicutes;c__Bacilli;o__Lactobacillales;f__Lactobacillaceae;g__Lactobacillus	0.00	0.00	0.00	0.00	0.10	0.00	0.00	0.00	0.00
Firmicutes;c__Bacilli;o__Paenibacillales;f__Paenibacillaceae;g__Paenibacillus	0.07	0.00	0.00	0.00	0.00	0.00	0.00	0.00	0.00
Firmicutes;c__Bacilli;o__Thermoactinomycetales;f__Thermoactinomycetaceae;g__Planifilum	0.00	0.00	0.00	0.00	0.19	0.00	0.00	0.00	0.00
Firmicutes;c__Clostridia;o__Clostridiales;f__Clostridiaceae	0.10	0.00	0.00	0.00	0.00	0.00	0.00	0.00	0.00
Gemmatimonadota;c__Gemmatimonadetes;o__Gemmatimonadales;f__Gemmatimonadaceae	0.14	0.00	0.00	0.00	0.00	0.00	0.00	0.00	0.00
Gemmatimonadota;c__Gemmatimonadetes;o__Gemmatimonadales;f__Gemmatimonadaceae;g__Gemmatimonas	0.64	0.01	0.02	0.00	0.00	0.00	0.00	0.00	0.00
Gemmatimonadota;c__Gemmatimonadetes;o__Gemmatimonadales;f__Gemmatimonadaceae;g__uncultured	0.49	0.02	0.00	0.00	0.00	0.00	0.00	0.00	0.00
Latescibacterota;c__Latescibacterota;o__Latescibacterota;f__Latescibacterota;g__Latescibacterota	0.96	0.00	0.00	0.00	0.00	0.00	0.00	0.00	0.00
Methylomirabilota;c__Methylomirabilia;o__Rokubacteriales;f__Rokubacteriales;g__Rokubacteriales	0.16	0.00	0.00	0.00	0.00	0.00	0.00	0.00	0.00
Methylomirabilota;c__Methylomirabilia;o__Rokubacteriales;f__WX65;g__WX65	0.33	0.00	0.00	0.00	0.00	0.00	0.00	0.00	0.00
Myxococcota;c__Myxococcia;o__Myxococcales;f__Anaeromyxobacteraceae;g__Anaeromyxobacter	0.17	0.00	0.00	0.00	0.00	0.00	0.00	0.00	0.00
Myxococcota;c__Myxococcia;o__Myxococcales;f__Myxococcaceae	0.14	0.00	0.00	0.00	0.00	0.00	0.00	0.00	0.00
Myxococcota;c__Myxococcia;o__Myxococcales;f__Myxococcaceae;g__P3OB-42	0.08	0.00	0.00	0.00	0.00	0.00	0.00	0.00	0.00
Myxococcota;c__Myxococcia;o__Myxococcales;f__Myxococcaceae;g__uncultured	0.18	0.00	0.00	0.00	0.00	0.00	0.00	0.00	0.00
Myxococcota;c__Polyangia;o__Haliangiales;f__Haliangiaceae;g__Haliangium	0.84	0.00	0.00	0.00	0.00	0.00	0.00	0.00	0.00
Myxococcota;c__Polyangia;o__Polyangiales;f__BIrii41;g__BIrii41	0.13	0.00	0.00	0.00	0.00	0.00	0.00	0.00	0.00
Myxococcota;c__Polyangia;o__Polyangiales;f__Phaselicystidaceae;g__Phaselicystis	0.22	0.00	0.00	0.00	0.00	0.00	0.00	0.00	0.00
Myxococcota;c__Polyangia;o__Polyangiales;f__Polyangiaceae;g__Pajaroellobacter	0.16	0.00	0.00	0.00	0.00	0.00	0.00	0.00	0.00
Nitrospirota;c__Nitrospiria;o__Nitrospirales;f__Nitrospiraceae;g__Nitrospira	0.36	0.00	0.00	0.00	0.00	0.00	0.00	0.00	0.00
Patescibacteria;c__Saccharimonadia;o__Saccharimonadales;f__Saccharimonadales;g__Saccharimonadales	0.08	0.00	0.05	0.00	0.00	0.00	0.01	0.00	0.00
Planctomycetota;c__Planctomycetes;o__Gemmatales;f__Gemmataceae	0.17	0.00	0.00	0.00	0.00	0.00	0.00	0.00	0.00
Planctomycetota;c__Planctomycetes;o__Gemmatales;f__Gemmataceae;g__Fimbriiglobus	0.06	0.00	0.00	0.00	0.00	0.00	0.00	0.00	0.00
Planctomycetota;c__Planctomycetes;o__Gemmatales;f__Gemmataceae;g__Gemmata	0.29	0.00	0.00	0.00	0.00	0.00	0.00	0.00	0.00
Planctomycetota;c__Planctomycetes;o__Gemmatales;f__Gemmataceae;g__Telmatocola	0.09	0.03	0.00	0.00	0.00	0.00	0.00	0.00	0.00
Planctomycetota;c__Planctomycetes;o__Gemmatales;f__Gemmataceae;g__uncultured	0.86	0.01	0.00	0.08	0.00	0.05	0.00	0.00	0.00
Planctomycetota;c__Planctomycetes;o__Isosphaerales;f__Isosphaeraceae	0.37	0.00	0.00	0.00	0.00	0.00	0.00	0.00	0.00
Planctomycetota;c__Planctomycetes;o__Pirellulales;f__Pirellulaceae	0.08	0.00	0.00	0.02	0.00	0.00	0.00	0.00	0.00
Planctomycetota;c__Planctomycetes;o__Pirellulales;f__Pirellulaceae;g__Pir4_lineage	0.10	0.00	0.00	0.00	0.00	0.00	0.00	0.00	0.00
Planctomycetota;c__Planctomycetes;o__Pirellulales;f__Pirellulaceae;g__Pirellula	0.17	0.01	0.00	0.00	0.00	0.07	0.00	0.00	0.00
Planctomycetota;c__Planctomycetes;o__Planctomycetales;f__Schlesneriaceae;g__Schlesneria	0.00	0.00	0.00	0.00	0.00	0.00	0.00	0.00	0.00
Planctomycetota;c__Planctomycetes;o__Planctomycetales;f__uncultured;g__uncultured	0.18	0.00	0.02	0.00	0.00	0.00	0.00	0.00	0.00
Proteobacteria;c__Alphaproteobacteria	0.51	0.00	0.10	0.12	0.00	0.00	0.00	0.00	0.00
Proteobacteria;c__Alphaproteobacteria;o__Acetobacterales;f__Acetobacteraceae	0.15	0.07	0.10	0.08	0.00	0.00	0.02	0.06	0.00
Proteobacteria;c__Alphaproteobacteria;o__Azospirillales;f__Inquilinaceae;g__Inquilinus	0.00	0.02	0.00	0.00	0.00	0.12	0.02	0.00	0.00
Proteobacteria;c__Alphaproteobacteria;o__Azospirillales;f__uncultured;g__uncultured	0.13	0.00	0.00	0.00	0.00	0.00	0.00	0.00	0.00
Proteobacteria;c__Alphaproteobacteria;o__Caulobacterales;f__Caulobacteraceae	0.02	0.99	1.38	0.20	0.10	0.37	0.00	0.39	0.05
Proteobacteria;c__Alphaproteobacteria;o__Caulobacterales;f__Caulobacteraceae;g__Phenylobacterium	0.10	0.34	0.12	0.11	0.17	0.00	0.00	0.00	0.00
Proteobacteria;c__Alphaproteobacteria;o__Caulobacterales;f__Caulobacteraceae;g__uncultured	0.02	0.08	0.03	0.00	0.00	0.00	0.00	0.00	0.00
Proteobacteria;c__Alphaproteobacteria;o__Defluviicoccales;f__Defluviicoccaceae;g__Defluviicoccus	0.13	0.00	0.00	0.00	0.00	0.00	0.00	0.00	0.00
Proteobacteria;c__Alphaproteobacteria;o__Dongiales;f__Dongiaceae;g__Dongia	0.18	0.02	0.00	0.00	0.00	0.00	0.00	0.00	0.00
Proteobacteria;c__Alphaproteobacteria;o__Elsterales;f__uncultured;g__uncultured	0.03	0.01	0.06	0.02	0.00	0.00	0.02	0.00	0.00
Proteobacteria;c__Alphaproteobacteria;o__Reyranellales;f__Reyranellaceae;g__Reyranella	0.73	0.59	0.58	0.28	0.03	0.79	0.13	0.00	0.04
Proteobacteria;c__Alphaproteobacteria;o__Rhizobiales	0.21	0.22	0.00	0.11	0.00	0.21	0.05	0.04	0.00
Proteobacteria;c__Alphaproteobacteria;o__Rhizobiales;f__Beijerinckiaceae	0.17	0.02	0.02	0.04	0.00	0.00	0.06	0.00	0.00
Proteobacteria;c__Alphaproteobacteria;o__Rhizobiales;f__Beijerinckiaceae;g__Methylobacterium-Methylorubrum	0.01	0.22	0.03	0.00	0.07	0.16	0.00	0.00	0.00
Proteobacteria;c__Alphaproteobacteria;o__Rhizobiales;f__Devosiaceae	0.00	0.09	0.06	0.07	0.05	0.37	0.24	0.01	0.08
Proteobacteria;c__Alphaproteobacteria;o__Rhizobiales;f__Devosiaceae;g__Devosia	0.02	0.09	0.10	0.00	0.00	0.00	0.00	0.06	0.03
Proteobacteria;c__Alphaproteobacteria;o__Rhizobiales;f__Hyphomicrobiaceae	0.31	0.00	0.00	0.00	0.00	0.00	0.00	0.00	0.00
Proteobacteria;c__Alphaproteobacteria;o__Rhizobiales;f__Hyphomicrobiaceae;g__Pedomicrobium	0.42	0.00	0.00	0.00	0.00	0.00	0.00	0.00	0.00
Proteobacteria;c__Alphaproteobacteria;o__Rhizobiales;f__Labraceae;g__Labrys	0.00	0.00	0.00	0.07	0.00	0.00	0.01	0.00	0.02
Proteobacteria;c__Alphaproteobacteria;o__Rhizobiales;f__Methyloligellaceae;g__Methyloceanibacter	0.71	0.00	0.00	0.00	0.00	0.00	0.00	0.00	0.00
Proteobacteria;c__Alphaproteobacteria;o__Rhizobiales;f__Methyloligellaceae;g__uncultured	1.64	0.00	0.00	0.00	0.00	0.00	0.00	0.00	0.00
Proteobacteria;c__Alphaproteobacteria;o__Rhizobiales;f__Rhizobiaceae	0.34	0.38	0.70	0.42	0.00	1.36	2.26	0.56	0.07
Proteobacteria;c__Alphaproteobacteria;o__Rhizobiales;f__Rhizobiaceae;g__Allorhizobium-Neorhizobium-Pararhizobium-Rhiz	0.08	1.20	1.61	3.44	0.00	7.18	6.65	0.76	0.54
Proteobacteria;c__Alphaproteobacteria;o__Rhizobiales;f__Rhizobiales_Incertae_Sedis;g__Nordella	0.12	0.00	0.00	0.00	0.00	0.00	0.00	0.00	0.00
Proteobacteria;c__Alphaproteobacteria;o__Rhizobiales;f__Rhodomicrobiaceae;g__Rhodomicrobium	0.15	0.00	0.00	0.00	0.00	0.00	0.00	0.00	0.00
Proteobacteria;c__Alphaproteobacteria;o__Rhizobiales;f__Xanthobacteraceae	1.05	2.12	1.96	2.78	5.24	5.04	1.96	2.13	1.43
Proteobacteria;c__Alphaproteobacteria;o__Rhizobiales;f__Xanthobacteraceae	9.39	2.51	2.31	3.24	5.62	5.64	2.31	2.28	1.66
Proteobacteria;c__Alphaproteobacteria;o__Rhizobiales;f__Xanthobacteraceae;g__Bradyrhizobium	0.90	0.37	0.26	0.40	0.38	0.44	0.29	0.15	0.21
Proteobacteria;c__Alphaproteobacteria;o__Rhizobiales;f__Xanthobacteraceae;g__Pseudolabrys	0.03	0.00	0.00	0.03	0.00	0.00	0.06	0.00	0.00
Proteobacteria;c__Alphaproteobacteria;o__Rhizobiales;f__Xanthobacteraceae;g__Rhodoplanes	0.33	0.02	0.00	0.00	0.00	0.00	0.00	0.00	0.00
Proteobacteria;c__Alphaproteobacteria;o__Rhizobiales;f__Xanthobacteraceae;g__uncultured	7.08	0.00	0.10	0.03	0.00	0.16	0.00	0.00	0.02
Proteobacteria;c__Alphaproteobacteria;o__Rhodobacterales;f__Rhodobacteraceae;g__Rhodobacter	0.00	0.05	0.00	0.09	0.00	0.00	0.01	0.00	0.06
Proteobacteria;c__Alphaproteobacteria;o__Sphingomonadales;f__Sphingomonadaceae	0.40	40.27	35.67	49.32	90.78	28.67	15.85	6.55	18.00
Proteobacteria;c__Alphaproteobacteria;o__Sphingomonadales;f__Sphingomonadaceae;g__Novosphingobium	0.00	0.74	0.62	0.17	0.07	1.22	0.01	0.06	0.57
Proteobacteria;c__Alphaproteobacteria;o__Sphingomonadales;f__Sphingomonadaceae;g__Sphingobium	0.00	29.03	15.66	16.69	0.34	13.03	4.23	3.12	1.40
Proteobacteria;c__Alphaproteobacteria;o__Sphingomonadales;f__Sphingomonadaceae;g__Sphingomonas	0.06	2.91	1.48	1.48	0.05	0.35	0.17	0.37	2.02
Proteobacteria;c__Alphaproteobacteria;o__Tistrellales;f__Geminicoccaceae;g__Candidatus_Alysiosphaera	0.11	0.00	0.00	0.00	0.00	0.00	0.00	0.00	0.00
Proteobacteria;c__Alphaproteobacteria;o__uncultured;f__uncultured;g__uncultured	0.15	0.00	0.03	0.00	0.00	0.00	0.00	0.00	0.00
Proteobacteria;c__Gammaproteobacteria	0.33	0.03	0.02	0.00	0.00	0.00	0.00	0.00	0.00
Proteobacteria;c__Gammaproteobacteria;o__Burkholderiales	0.24	0.00	0.00	0.00	0.00	0.00	0.00	0.00	0.00
Proteobacteria;c__Gammaproteobacteria;o__Burkholderiales;f__A21b;g__A21b	0.22	0.00	0.00	0.00	0.00	0.00	0.00	0.00	0.00
Proteobacteria;c__Gammaproteobacteria;o__Burkholderiales;f__Burkholderiaceae	0.06	0.82	1.27	0.24	0.13	2.90	0.61	0.51	0.22
Proteobacteria;c__Gammaproteobacteria;o__Burkholderiales;f__Burkholderiaceae;g__Burkholderia-Caballeronia-Paraburkho	0.04	0.25	0.17	0.11	0.00	2.00	0.26	0.13	0.06
Proteobacteria;c__Gammaproteobacteria;o__Burkholderiales;f__Burkholderiaceae;g__Cupriavidus	0.02	0.21	0.72	0.07	0.06	0.16	0.26	0.23	0.16
Proteobacteria;c__Gammaproteobacteria;o__Burkholderiales;f__Burkholderiaceae;g__Ralstonia	0.00	0.36	0.38	0.06	0.08	0.73	0.09	0.16	0.00
Proteobacteria;c__Gammaproteobacteria;o__Burkholderiales;f__Comamonadaceae	0.14	0.52	0.64	0.14	0.05	0.05	0.06	0.10	0.01
Proteobacteria;c__Gammaproteobacteria;o__Burkholderiales;f__Comamonadaceae;g__Ramlibacter	0.05	0.03	0.03	0.00	0.00	0.09	0.00	0.05	0.00
Proteobacteria;c__Gammaproteobacteria;o__Burkholderiales;f__Comamonadaceae;g__Rhodoferax	0.00	0.53	0.17	0.39	0.00	0.07	0.00	0.55	0.06
Proteobacteria;c__Gammaproteobacteria;o__Burkholderiales;f__Comamonadaceae;g__Rubrivivax	0.04	0.05	0.03	0.02	0.00	0.00	0.00	0.00	0.00
Proteobacteria;c__Gammaproteobacteria;o__Burkholderiales;f__Comamonadaceae;g__Variovorax	0.02	1.54	0.80	0.02	0.00	0.79	0.13	0.55	0.21
Proteobacteria;c__Gammaproteobacteria;o__Burkholderiales;f__Nitrosomonadaceae;g__Ellin6067	0.40	0.00	0.05	0.00	0.00	0.00	0.00	0.00	0.00
Proteobacteria;c__Gammaproteobacteria;o__Burkholderiales;f__Nitrosomonadaceae;g__mle1-7	0.21	0.00	0.00	0.00	0.00	0.00	0.00	0.00	0.00
Proteobacteria;c__Gammaproteobacteria;o__Burkholderiales;f__Nitrosomonadaceae;g__MND1	0.40	0.00	0.02	0.00	0.00	0.00	0.00	0.00	0.00
Proteobacteria;c__Gammaproteobacteria;o__Burkholderiales;f__SC-I-84;g__SC-I-84	0.92	0.00	0.00	0.00	0.00	0.00	0.00	0.00	0.00
Proteobacteria;c__Gammaproteobacteria;o__CCD24;f__CCD24;g__CCD24	0.14	0.00	0.00	0.00	0.00	0.00	0.00	0.00	0.00
Proteobacteria;c__Gammaproteobacteria;o__Diplorickettsiales;f__Diplorickettsiaceae;g__Aquicella	0.09	0.00	0.00	0.00	0.01	0.00	0.00	0.00	0.00
Proteobacteria;c__Gammaproteobacteria;o__Diplorickettsiales;f__Diplorickettsiaceae;g__uncultured	0.06	0.02	0.00	0.03	0.00	0.00	0.00	0.00	0.00
Proteobacteria;c__Gammaproteobacteria;o__Enterobacterales	0.00	0.05	0.07	0.83	0.00	0.00	0.71	0.04	0.06
Proteobacteria;c__Gammaproteobacteria;o__Enterobacterales;f__Enterobacteriaceae	0.00	0.23	0.12	0.20	0.07	0.33	3.08	0.00	0.28
Proteobacteria;c__Gammaproteobacteria;o__Enterobacterales;f__Enterobacteriaceae	0.00	0.26	0.12	1.43	0.07	0.73	4.50	0.05	0.32
Proteobacteria;c__Gammaproteobacteria;o__Enterobacterales;f__Enterobacteriaceae;g__Enterobacter	0.00	0.00	0.00	0.13	0.00	0.00	0.37	0.05	0.00
Proteobacteria;c__Gammaproteobacteria;o__Enterobacterales;f__Erwiniaceae	0.00	0.03	0.00	0.97	0.00	0.40	1.01	0.00	0.04
Proteobacteria;c__Gammaproteobacteria;o__Enterobacterales;f__Erwiniaceae;g__Erwinia	0.00	0.00	0.00	0.14	0.00	0.00	0.03	0.00	0.00
Proteobacteria;c__Gammaproteobacteria;o__Enterobacterales;f__Erwiniaceae;g__Pantoea	0.00	0.06	0.26	3.10	0.00	0.24	0.26	0.00	0.00
Proteobacteria;c__Gammaproteobacteria;o__Enterobacterales;f__Yersiniaceae;g__Rahnella1	0.00	0.00	0.01	1.20	0.00	0.98	0.90	0.14	0.32
Proteobacteria;c__Gammaproteobacteria;o__Enterobacterales;f__Yersiniaceae;g__Serratia	0.00	0.00	0.01	0.74	0.00	0.00	0.16	0.00	0.00
Proteobacteria;c__Gammaproteobacteria;o__Gammaproteobacteria_Incertae_Sedis;f__Unknown_Family;g__Acidibacter	0.21	0.00	0.00	0.00	0.00	0.00	0.00	0.00	0.00
Proteobacteria;c__Gammaproteobacteria;o__Pseudomonadales;f__Moraxellaceae;g__Acinetobacter	0.00	0.05	0.06	0.03	0.00	0.05	0.00	0.58	0.03
Proteobacteria;c__Gammaproteobacteria;o__Pseudomonadales;f__Pseudomonadaceae;g__Pseudomonas	0.06	2.97	5.82	2.25	0.02	3.27	24.48	69.56	60.16
Proteobacteria;c__Gammaproteobacteria;o__Steroidobacterales;f__Steroidobacteraceae;g__Steroidobacter	0.18	0.00	0.00	0.00	0.00	0.00	0.00	0.00	0.00
Proteobacteria;c__Gammaproteobacteria;o__Steroidobacterales;f__Steroidobacteraceae;g__uncultured	0.10	0.00	0.00	0.00	0.00	0.00	0.00	0.00	0.00
Proteobacteria;c__Gammaproteobacteria;o__Xanthomonadales;f__Xanthomonadaceae;g__Stenotrophomonas	0.00	0.10	0.04	0.02	0.00	0.04	0.00	0.34	0.03
Verrucomicrobiota;c__Verrucomicrobiae;o__Chthoniobacterales	1.85	0.00	0.00	0.00	0.00	0.00	0.00	0.00	0.02
Verrucomicrobiota;c__Verrucomicrobiae;o__Chthoniobacterales;f__Chthoniobacteraceae;g__Candidatus_Udaeobacter	9.39	0.00	0.00	0.00	0.00	0.00	0.00	0.00	0.00
Verrucomicrobiota;c__Verrucomicrobiae;o__Chthoniobacterales;f__Chthoniobacteraceae;g__Chthoniobacter	0.32	0.20	0.02	0.08	0.00	0.05	0.04	0.08	0.07
Verrucomicrobiota;c__Verrucomicrobiae;o__Chthoniobacterales;f__Terrimicrobiaceae;g__Terrimicrobium	0.24	0.00	0.00	0.00	0.00	0.15	0.00	0.00	0.01
Verrucomicrobiota;c__Verrucomicrobiae;o__Chthoniobacterales;f__Xiphinematobacteraceae;g__Candidatus_Xiphinematoba	0.64	0.04	0.00	0.00	0.00	0.05	0.00	0.04	0.00
Verrucomicrobiota;c__Verrucomicrobiae;o__Opitutales;f__Opitutaceae	0.09	0.06	0.00	0.01	0.00	0.00	0.00	0.00	0.00
Verrucomicrobiota;c__Verrucomicrobiae;o__Pedosphaerales;f__Pedosphaeraceae	0.30	0.00	0.00	0.00	0.00	0.00	0.00	0.00	0.00
Verrucomicrobiota;c__Verrucomicrobiae;o__Pedosphaerales;f__Pedosphaeraceae;g__ADurb.Bin063-1	0.63	0.00	0.00	0.00	0.00	0.00	0.00	0.00	0.00
Verrucomicrobiota;c__Verrucomicrobiae;o__Pedosphaerales;f__Pedosphaeraceae;g__Pedosphaeraceae	0.58	0.00	0.00	0.00	0.00	0.00	0.00	0.00	0.00
Verrucomicrobiota;c__Verrucomicrobiae;o__Pedosphaerales;f__Pedosphaeraceae;g__uncultured	0.26	0.00	0.00	0.00	0.00	0.00	0.00	0.00	0.00
Verrucomicrobiota;c__Verrucomicrobiae;o__Verrucomicrobiales;f__Rubritaleaceae;g__Luteolibacter	0.05	0.00	0.00	0.00	0.00	0.00	0.00	0.00	0.15
Verrucomicrobiota;c__Verrucomicrobiae;o__Verrucomicrobiales;f__Verrucomicrobiaceae	0.01	0.08	0.00	0.00	0.00	0.00	0.00	0.00	0.14
Verrucomicrobiota;c__Verrucomicrobiae;o__Verrucomicrobiales;f__Verrucomicrobiaceae;g__Roseimicrobium	0.12	0.05	0.00	0.12	0.00	0.08	0.00	0.01	0.01
Verrucomicrobiota;c__Verrucomicrobiae;o__Verrucomicrobiales;f__Verrucomicrobiaceae;g__uncultured	0.03	0.03	0.00	0.00	0.00	0.00	0.00	0.00	0.14
d__Archaea;Crenarchaeota;c__Nitrososphaeria;o__Nitrososphaerales;f__Nitrososphaeraceae;g__Nitrososphaeraceae	1.16	0.00	0.00	0.00	0.00	0.00	0.00	0.00	0.00

Relative taxon abundance in Arctic core samples and in permafrost populations sorted follwing BONCAT incubations.

D1, active layer; D3, permafrost; G, sorted based on A488 click label; N, incubation with exogenous nutrients

Taxon	D1	D3	D3_6w G	D3_6wN G
Acidobacteriota;c__Holophagae;o__Subgroup_7;f__Subgroup_7;g__Subgroup_7	1.16	0.00	0.00	0.00
Acidobacteriota;c__Vicinamibacteria;o__Vicinamibacterales;f__uncultured;g__uncultured	7.48	0.00	0.00	0.00
Acidobacteriota;c__Vicinamibacteria;o__Vicinamibacterales;f__Vicinamibacteraceae;g__Vicinamibacteraceae	0.93	0.00	0.00	0.00
Actinobacteriota;c__Acidimicrobiia;o__IMCC26256;f__IMCC26256;g__IMCC26256	0.87	0.77	0.00	0.00
Actinobacteriota;c__Acidimicrobiia;o__Microtrichales;f__Ilumatobacteraceae;g__CL500-29_marine_group	3.13	0.00	0.00	0.00
Actinobacteriota;c__Actinobacteria;o__0319-7L14;f__0319-7L14;g__0319-7L14	0.00	6.12	0.00	0.00
Actinobacteriota;c__Actinobacteria;o__Corynebacteriales;f__Corynebacteriaceae;g__Corynebacterium	0.23	1.01	0.00	0.00
Actinobacteriota;c__Actinobacteria;o__Corynebacteriales;f__Corynebacteriaceae;g__Lawsonella	0.00	2.35	0.00	0.03
Actinobacteriota;c__Actinobacteria;o__Corynebacteriales;f__Nocardiaceae;g__Rhodococcus	0.44	1.84	59.13	31.60
Actinobacteriota;c__Actinobacteria;o__Micrococcales;f__Microbacteriaceae;__	0.00	0.00	0.00	0.45
Actinobacteriota;c__Actinobacteria;o__Micrococcales;f__Microbacteriaceae;g__Leifsonia	0.00	0.00	0.00	0.00
Actinobacteriota;c__Actinobacteria;o__Micrococcales;f__Micrococcaceae;__	1.30	0.00	0.00	6.31
Actinobacteriota;c__Actinobacteria;o__Micrococcales;f__Micrococcaceae;g__Pseudarthrobacter	4.46	0.00	0.00	0.00
Actinobacteriota;c__Thermoleophilia;o__Gaiellales;f__Gaiellaceae;g__Gaiella	1.81	0.00	0.00	0.00
Actinobacteriota;c__Thermoleophilia;o__Gaiellales;f__uncultured;g__uncultured	2.79	1.00	0.00	0.00
Bacteroidota;c__Bacteroidia;o__Cytophagales;f__Hymenobacteraceae;g__Hymenobacter	0.00	0.00	0.00	0.00
Bacteroidota;c__Bacteroidia;o__Cytophagales;f__Spirosomaceae;g__Dyadobacter	0.00	0.00	0.00	0.00
Bacteroidota;c__Bacteroidia;o__Flavobacteriales;f__Flavobacteriaceae;g__Flavobacterium	0.02	0.00	1.03	0.00
Bacteroidota;c__Bacteroidia;o__Sphingobacteriales;f__Sphingobacteriaceae;g__Mucilaginibacter	0.00	0.00	0.26	0.00
Chloroflexi;c__Chloroflexia;o__Thermomicrobiales;f__JG30-KF-CM45;g__JG30-KF-CM45	0.07	0.00	0.00	0.00
Chloroflexi;c__Gitt-GS-136;o__Gitt-GS-136;f__Gitt-GS-136;g__Gitt-GS-136	2.60	0.00	0.00	0.00
Chloroflexi;c__KD4-96;o__KD4-96;f__KD4-96;g__KD4-96	4.90	0.00	0.00	0.00
Chloroflexi;c__P2-11E;o__P2-11E;f__P2-11E;g__P2-11E	0.86	0.59	0.00	0.00
Firmicutes;c__Bacilli;o__Paenibacillales;f__Paenibacillaceae;g__Paenibacillus	0.00	0.02	0.00	0.93
Gemmatimonadota;c__Gemmatimonadetes;o__Gemmatimonadales;f__Gemmatimonadaceae;g__uncultured	1.56	0.00	0.00	0.00
Myxococcota;c__Polyangia;o__Nannocystales;f__Nannocystaceae;g__uncultured	0.00	2.76	0.00	0.00
Proteobacteria;c__Alphaproteobacteria;o__Rhizobiales;f__Rhizobiaceae;g__Aliihoeflea	0.00	0.00	20.31	18.45
Proteobacteria;c__Alphaproteobacteria;o__Rhizobiales;f__Rhizobiaceae;g__Aureimonas	0.00	0.70	3.74	10.24
Proteobacteria;c__Alphaproteobacteria;o__Rhizobiales;f__Rhizobiaceae;g__Phyllobacterium	0.61	47.48	0.00	0.00
Proteobacteria;c__Alphaproteobacteria;o__Rhizobiales;f__Xanthobacteraceae;g__Bradyrhizobium	0.35	2.32	0.00	0.00
Proteobacteria;c__Alphaproteobacteria;o__Rhizobiales;f__Xanthobacteraceae;g__uncultured	11.89	0.00	0.00	0.00
Proteobacteria;c__Alphaproteobacteria;o__Sphingomonadales;f__Sphingomonadaceae;g__Sphingomonas	19.46	0.00	0.08	2.83
Proteobacteria;c__Gammaproteobacteria;o__Burkholderiales;f__Comamonadaceae;g__Variovorax	0.04	0.12	1.85	5.33
Proteobacteria;c__Gammaproteobacteria;o__Burkholderiales;f__Oxalobacteraceae;g__Massilia	0.00	1.33	7.43	0.00
Proteobacteria;c__Gammaproteobacteria;o__Pseudomonadales;f__Moraxellaceae;g__Enhydrobacter	0.00	3.60	0.00	0.00
Proteobacteria;c__Gammaproteobacteria;o__Pseudomonadales;f__Moraxellaceae;g__Psychrobacter	0.00	0.00	1.16	21.93
Proteobacteria;c__Gammaproteobacteria;o__Pseudomonadales;f__Pseudomonadaceae;g__Pseudomonas	0.79	9.47	2.97	0.08
Verrucomicrobiota;c__Verrucomicrobiae;o__Chthoniobacterales;f__Chthoniobacteraceae;g__Candidatus_Udaeobacter	1.42	0.00	0.00	0.00
WPS-2;c__WPS-2;o__WPS-2;f__WPS-2;g__WPS-2	4.63	0.00	0.00	0.00

Relative major genera/families abundance in oral sample and in populations sorted follwing BONCAT incubations.

R, sorted based on red fluoresence (DNA stain). G, sorted based on A488 click label

Description	Oral	MTGE R	MTGE G	MTGE High G	Dextrose R	Dextrose G	Lactate G	Lactate High G	NAG G
Actinobacteriota_Actinomyces	2.98	0.05	0.13	0.05	0.38	0.24	0.18	0.03	0.34
Actinobacteriota_Scardovia	0.00	0.00	0.00	0.00	0.00	0.01	0.00	0.00	0.00
Actinobacteriota_Corynebacterium	0.41	0.00	0.00	0.00	0.03	0.02	0.01	0.01	0.00
Actinobacteriota_Atopobiaceae	0.01	0.46	0.83	0.57	5.79	8.78	6.46	4.60	5.94
Bacteroidota_Paludibacteraceae	0.00	0.15	0.04	0.33	0.55	0.77	0.32	2.71	0.23
Bacteroidota_Porphyromonas	1.07	0.10	0.61	0.13	0.96	1.14	0.62	0.24	0.71
Bacteroidota_Alloprevotella	6.09	0.48	1.15	1.69	0.58	0.69	0.49	0.28	0.85
Bacteroidota_Prevotella	19.97	2.56	4.08	5.92	9.50	10.61	7.57	15.43	9.57
Bacteroidota_Capnocytophaga	0.58	0.18	0.89	0.90	3.07	2.96	1.96	1.70	1.83
Bacteroidota_Bergeyella	0.00	0.29	0.01	0.51	0.76	1.44	0.52	5.63	0.28
Campilobacterota_Campylobacter	0.40	0.56	0.64	0.90	3.30	3.37	0.90	1.97	1.11
Firmicutes_Gemella	3.04	7.85	25.30	9.20	3.27	1.59	1.34	0.30	2.73
Firmicutes_Granulicatella	3.48	0.26	1.07	0.39	0.24	0.24	0.07	0.00	0.29
Firmicutes_Streptococcus	28.46	16.16	15.60	10.43	7.55	8.73	3.25	1.69	14.29
Firmicutes_Lachnospiraceae	1.88	6.30	0.74	3.66	1.77	3.86	0.98	5.89	2.35
Firmicutes_Peptostreptococcus	0.64	7.34	6.73	8.94	8.31	2.27	0.40	0.12	0.32
Firmicutes_Selenomonas	0.09	0.35	0.25	0.51	1.44	1.53	2.44	1.89	4.74
Firmicutes_Veillonella	7.59	17.45	22.40	7.94	16.28	6.87	56.48	11.84	28.66
Fusobacteriota_Fusobacterium	2.63	2.93	1.91	2.08	12.44	20.91	3.57	5.07	6.62
Fusobacteriota_Leptotrichia	1.29	24.74	0.43	20.48	3.51	3.82	1.03	9.46	2.70
Patescibacteria_Saccharimonadaceae	0.71	0.43	0.33	0.33	2.50	4.26	1.75	6.23	1.74
Gammaproteobacteria_Neisseria	2.57	0.00	0.02	0.00	0.43	0.28	0.26	0.13	0.22
Gammaproteobacteria_Pasteurellaceae	10.46	7.37	13.17	8.93	6.06	4.01	1.77	4.65	2.78
